# ﻿A protocol to evaluate the taxonomic health of Neotropical species of *Nasutitermes* (Termitidae, Nasutitermitinae)

**DOI:** 10.3897/zookeys.1210.116666

**Published:** 2024-08-22

**Authors:** Mauricio M. Rocha, Karina S. S. Lima, Eliana M. Cancello

**Affiliations:** 1 Museu de Zoologia da Universidade de São Paulo, São Paulo, SP, Brazil Museu de Zoologia da Universidade de São Paulo São Paulo Brazil

**Keywords:** Collections, revisionary work, rich taxa, species description, taxonomic protocol, taxonomic ranking

## Abstract

Herein a protocol is proposed to summarize the taxonomic situation for species, using the Neotropical *Nasutitermes* Dudley (Nasutitermitinae) as a test. The objective of this protocol is to allow comparisons between the available taxonomic information for species, and to provide objective criteria for assessing the information gaps for each taxon in order to prioritize topics for future investigation. Key aspects of taxonomic practice (condition of type specimens, helpfulness of descriptions and figures, compilation of distribution data, molecular data) were noted, the data were tabulated, and the taxa ranked. In addition, specific notes for each species have been included that may help to improve the solutions to the problems raised here.

## ﻿Introduction

Taxonomy as a science is misunderstood by most people, and worse yet, this includes many professional biologists, including those responsible for policy decisions and financial allocations. It is a descriptive science, like many others that are more easily recognized to many, such as astronomy with its stellar descriptions and particle physics with the description, albeit “high-tech”, of fundamental particles. Grimaldi and Engel (2007) made a vigorous defense of the comparative scientific approach for all descriptive disciplines, arguing against the “pejorative” view regarding the term descriptive in science.

There are many papers in defense of taxonomy and discussing the concept of a “Taxonomic Impediment” (e.g., de Carvalho et al. 2007; Engel et al. 2021), although some interpretations are equivocal, like the idea that the impediment can be solved merely with faster descriptions. Taxonomy is a scientific discipline based on hypotheses that may be challenged and tested using different tools, some are low-tech (anatomical and morphological studies) or “high-tech” (molecular data, nano-CT scans, etc.), and this distinction alone does not mean “more or less scientific”. All are equally valuable scientific approaches and each is good science to be considered on equal footing.

Taxonomy and nomenclature are not the same thing, they are different disciplines. Taxonomy describes species or higher taxa, searching for evolutionary lineages, that is, each species description is an evolutionary hypothesis. Nomenclature and its rules are used to achieve universal, consistent, and unambiguous communication among the scientific community. In this meaning, shared by all taxonomists, taxonomy is a science, whereas nomenclature is a technical discipline, an assemblage of rules to attribute names to organisms and their ranks.

Current taxonomy is not a “typological science”, as the types are merely a system by which the data of taxonomy are vouchered. Many biologists confuse these taxonomic vouchers as typological concepts simply because the word “type” is employed, but this is not the case at all. Nomenclatural types are used to link a name to one biological entity, and it does not “represent” this species. In this sense, it is fundamental to examine nomenclatural types, and when one is lost or damaged, this could be an actual problem to the attribution of that name. Names matter and confusion or misapplication of a name to a biological entity can have repercussions in everything from conservation and medicine to international treaties and national economies. A deeper discussion of scientific names and their importance and connection to good taxonomy was made by Engel (2022), from whom we copy here: “A lack of understanding that taxonomy and nomenclature are the foundation of all other biological disciplines has created a climate in which these sciences are dismissed and underfunded (Britz et al. 2020; Engel et al. 2021)”. An interesting and seminal book on practical taxonomic procedures by Winston (1999) is still exceptionally useful and should be consulted by all taxonomists and biologists.

A concise history of termite systematics was given by the treatise of Krishna et al. (2013). This 7-volume work covered all aspects of termite taxonomy up to that date, and the fifth volume was focused on the subfamilies Cubitermitinae and Nasutitermitinae, in with the species of the rich termite genus *Nasutitermes* Dudley occupying 164 of its 488 pages. *Nasutitermes* is the richest genus among termites and it is the type genus of the exceedingly rich subfamily Nasutitermitinae. Constantino (2002a) reviewed its fraught nomenclatural history, and Krishna et al. (2013) can be consulted for a detailed explanation.

*Nasutitermes* is one of the most frequently encountered termite genera in most Neotropical biome. Occupying a great distributional range across a remarkable variety of habitats, building different kinds of nests, and utilizing several food items, although most are xylophagous, some species are recognized as pests and others have the potential to become pestiferous. The group has evolved sophisticated defense strategies, with a large number of soldiers, equipped with effective chemical weapons. Despite the considerable biological and economic importance of species in the genus, the identification of species remains a great challenge, particularly as the genus comprises a vast number of described species, for which existing descriptions are mostly inadequate. As many species of *Nasutitermes* construct conspicuous nests, they were originally described by early entomologists and these antique scientific accounts have rarely been updated. Moreover, the existing data and descriptions are not uniform, sometimes they are exceedingly short and failed to include characters today considered of critical value, and in several cases the nomenclatural types were pinned instead of being preserved in alcohol, as is current best practice.

Holmgren (1906, 1910) described and redescribed many species of *Nasutitermes*. Although his descriptions are not uniform, he does mention head and nasus shapes and gives characters of the pilosity, which in the case of *Nasutitermes* may be helpful, as long as they are supplemented with other characters. His illustrations are simple and also not uniform as some of them show the pilosity, while others do not. In general, the head illustrations are effectively useless by modern standards. This author did describe the color, but this is now seldom helpful given that it is highly variable in most species. Because the measurements of head width and length are always present, if an expert considers all the characters together, and makes detailed comparison with other species, they can sometimes reach the right identification. Still, it is possible to suspect that there are many synonyms within *Nasutitermes*, and this situation is pointed out herein.

It is a consensus that the taxonomy of species of *Nasutitermes* is highly confusing and consequently the identification of its species is rendered difficult. The use of cuticular hydrocarbons was of some use to differentiate quite similar species, such as *N.corniger* and *N.ephratae* (Howard et al. 1988). Mitochondrial makers have demonstrated the synonymy of *N.corniger*, *N.costalis*, and *N.polygynus* (Scheffrahn et al. 2005a, 2005b). Pursuing the same goal, that is, to establish species delimitation among species of *Nasutitermes* from French Guiana, Roy et al. (2014) applied exploratory methods of species delimitation that use DNA sequences as their primary information source to establish group membership and estimate putative species boundaries. In the supplementary material of Roy et al. (2014) the authors explain how the species were named.

Gonzalez et al. (2013), with a very similar objective, utilized many of the criteria mentioned here (referred to as “taxonomic products”) to evaluate the taxonomic status of bumble bees in literature spanning from 1912 to 2011. They utilized Zoological Records and Web of Science, employing the search terms ‘bumble bees’. They categorized levels of operations (I to VIII), which represent combinations of taxonomic products [= diagnoses, descriptions, illustrations, keys, historical review, phylogeny, biogeography, molecular analyses] resulting from taxonomic work. Consequently, the levels of operation increase with the number of products combined. The paper offers more than just an example and should be referenced for a comprehensive understanding.

It is important to highlight that these criteria (their taxonomic products) are similar to our criteria, as they represent an expected standard in taxonomic work, arranged in a sequence, as mentioned there and proposed here. However, the protocol of Gonzalez et al. (2013) tries to evaluate the status of the literature/databases associated to a group of taxa, while our objective is to evaluate how robust the taxonomy associated to each taxon name is, in other words, the number and quality of elements/procedures of the taxonomic practice used to evaluate the species hypothesis represented by each name.

Kitchener et al. (2022) also explored a similar concept, aiming to establish a framework for assessing taxonomic certainty in mammals. They proposed a “traffic-light system” that integrates morphological, genetic, and biogeographical evidence, reflecting a conservation-oriented protocol.

Throughout the history of taxonomy, it took many decades for the concept of evolution to be more harshly incorporated by taxonomists (this is the reason why revisionary works are constantly relevant). Some groups were worked on by “excessively prolific” taxonomists, for example Maurice Pic, who by 1956 had described more than 18,500 beetle species, and was criticized by his contemporaries for his “chaotic methods”. Even today, with the use of modern techniques, some authors insist to propose uninformative species names. Some of these cases are discussed in Wheeler (2023), several associated with inappropriate use of molecular data.

The profusion of these “unsubstantiated names” creates difficulties to access information about biodiversity. Despite taxonomic vandalism cases, information about species, retrieved by their names, remains heterogeneous. For termites, Eggleton (1999) and Constantino (2018) conclude, by cumulative species description curves estimations, that the atypical high rates of description from China between 1980–2000 resulted in distorted estimates for the number of species expected for Oriental region. Similar problems occur in almost all groups of Hexapoda.

In face of the actual biodiversity crisis (Dirzo and Raven 2003; Pereira et al. 2010; Barnosky et al. 2011), much is discussed about species description rates (Bini et al. 2006; Dubois 2010; Mora et al. 2011). Although the lack of taxonomists is at the root of the taxonomic impediment, the problem is commonly misinterpreted as “slow species rate descriptions”, when the problem is also one of accessibility to names and associated information.

Considering these questions, we expect that this protocol can contribute to assess whether the names are a good reflection of the diversity of a group. Genera with many doubtful names “pollute” the information, artificially altering the perception of diversity, and are a problem. This is particularly relevant now that species databases are automatically generated by algorithms.

We also hope it will be useful for establishing priorities (among species and among their information) for updating knowledge. Some genera are very abundant in collections. Given the dynamics of how the research is practiced today, they will hardly be able to be completely revised. A very superficial estimate is that there are more than 12,000 samples of *Nasutitermes* in the major collections that cover the Neotropics, so it is necessary to establish priorities to work with its taxonomy. As a comparison, Sands (1965), in his review of the Ethiopian Nasutitermitinae, examined 2,400 samples.

In the case of *Nasutitermes*, one of the most frequently encountered and abundant genera in termite surveys, several names may prove problematic. However, assessing whether a genus has all its species names linked to robust taxonomic entities is also an interesting result. This information can be used to select taxa that are more appropriate targets for other studies, such as behavior or ecological approaches.

## ﻿Materials and methods

To evaluate the taxonomic health status of species, we developed a practical protocol that takes into account seven criteria applied to each species. Each criterion received a numeric value (elaborated in detail below). We used the following sources to evaluate each criterion: personal observations and information from institutional curators or collection managers; information compiled from the literature, referenced in Krishna et al. (2013); the Constantino Online termite database (Constantino 2023); and Google Scholar. In addition, we included any pertinent personal observations on each species (refer to Results, below).

The evaluation of the condition of type specimens was made via personal information [EMC visited the collections of the American Museum of Natural History (**AMNH**) and Naturhistoriska Riksmuseet (**NHRM**)], personal information from curators or collection managers [Museum of Comparative Zoology (**MCZ**), Natuurhistorisch Museum Maastricht (**NHMM**), and Zoologisches Museum für Hamburg (**ZMH**)], and online institutional databases.

### ﻿Explanation of the criteria

#### ﻿1 Type specimens

0 Lost or doubtful.

1 Badly conserved (e.g., pinned individuals).

2 Well preserved.

Many of the original descriptions of Isoptera were made before the broad implementation of the International Code of Zoological Nomenclature, so many species names are linked to syntype series. This practice may prove to be problematic when the type series includes more than one species.

Some of the first termite collectors used the old practice of preserving all insects on pins. Thus, some syntype series include pinned imagoes, workers, and soldiers, as exemplified in part by type material of Rambur in the Muséum national d’Histoire naturelle (**MNHN**, Paris) (EMC pers. obs.). In this kind of preservation, even if the specimen does not get broken, some relevant characters are impossible to examine due to the shrinkage of the soft integument.

#### ﻿2 Description based on

0 Imagoes and/or workers only

1 Soldiers only, soldiers and workers, or soldiers and imagoes

2 Soldiers, workers, and imagoes

Descriptions based on imagoes and/or workers are less useful for identification purposes, as the imagoes are uncommonly collected and congeneric workers recurrently have few distinctive characters. Moreover, for most species the imagoes are unknown, so descriptions associated with soldiers are much more useful. If the description includes worker characters (external sclerites and gut anatomy) this is a step that contributes to a more precise identification.

#### ﻿3 Description

0 Generic, i.e., do not allow species identification.

1 Clear, based on informative characters.

Species descriptions are not timeless: with the increasing number of described species, and more characters evaluated, old diagnoses tend to be relatively less informative and applicable. This does not mean that the author of the description was “unqualified”, simply that the description has become outmoded by the growth of biological information. All descriptions need to eventually be re-evaluated in the light of current data, methods, and concepts – hence the critical value of revisionary monographs.

The evaluation is not restricted to the first description; redescriptions and revisions may solve many species-identity problems and raise the rank. Some descriptions are wordy, with excessive generic characteristics that do not allow for meaningful identification (e.g., “nasus conical”).

#### ﻿Criteria used to evaluate a description as informative

- clear diagnostic characters mentioned in descriptions.
- inclusion of close congeneric species comparison.
- delimitation of the species through discrete characters.

A good species delimitation can be improved by quantitative characters (e.g., measurements), but this necessitates the availability of a large series of specimens and the evaluation of regional variations.

#### ﻿4 Description, illustrations

0 Illustrations/photographs missing or of poor quality.

1 With informative illustrations/photographs.

Illustrations and photographs that show clear informative characters are considered good quality. Even if present, some old illustrations are restricted to the soldier head silhouettes, which are not always useful.

#### ﻿5 Information about biology

0 Without information.

1 Some available data.

Data about nests can significantly increase identification rates since some species build quite particular nests or forage in specific substrates. In some cases, this information can solve a species identification, for example in *N.ephratae* and *N.corniger*, as reported by Thorne (1980). The soldiers have a similar morphology, while the nests exhibit typical features.

#### ﻿6 Data about species distribution

0 Known only from the type locality or sparse occurrences in the literature.

1 Partially compiled records.

2 The species occurrences are compiled and mapped.

Although some species of *Nasutitermes* are widespread (e.g., *N.corniger* occurs throughout South, Central, and insular America), several species have distributions correlated with environmental factors, and good knowledge about their distribution may be helpful for species discrimination and identification.

Records of species not based on vouchers in zoological collections have not been considered since such vouchers cannot be revised to identify potential misidentifications The same applies to citations of occurrences only by country, without georeferenced localities (checklists).

Partial records are distributions mapped based on records restricted a priori to a geographic vicinity (e.g., only to one country or only one biome). Papers with regional compilations have a more restricted utility to help in species identification but are better than the absence of information.

Well-mapped species distributions are a good indication that the species might have been properly revised.

#### ﻿7 Species delimited by DNA data

0 No data.

1 Species included in phylogenies or population-based studies.

DNA data here means that the species has been the subject of studies evaluating its identity and checking for species monophyly, rather than merely being a terminal included in an analysis or having sequences published or included in a DNA database. Examples are Scheffrahn et al. (2005a, 2005b) and Santos et al. (2017, 2022), which evaluated distinct populations.

### ﻿Taxonomic health ranks

The scores of each criterion are assessed for each species (Table 1); species are then ranked according to the sum of their individual scores. The taxonomic health of each species is classified into one of the following situations:

**Table 1.** Evaluation of criteria scores for each Neotropical species of *Nasutitermes* [1- Type specimens (0. Lost or doubtful, 1. Badly conserved, 2. Well-preserved); 2- Castes used for description (0. Imagoes and/or workers only, 1. Soldiers only, soldiers and workers, or soldiers and imagoes, 2. Soldiers, workers, and imagoes); 3- Description (0. Generic, not allowing species identification, 1. Clear, based on informative characters); 4- Description, illustrations (0. Illustrations/photos missing or of poor quality, 1. With informative illustrations/photos); 5- Information about biology (0. Without information, 1. Some available data); 6- Data about species distribution (0. Known only from the type locality or sparse records in the literature, 1. Partially compiled records, 2. The species registries are compiled and mapped); 7- Species delimited by DNA data (0. No data, 1. Species included in phylogenies or population studies)].

Species	1	2	3	4	5	6	7	Score
*Nasutitermeslividus*	1	0	0	0	0	0	0	1
*Nasutitermesfeytaudi*	2	0	0	0	0	0	0	2
*Nasutitermesmeinerti*	1	1	0	0	0	0	0	2
*Nasutitermesmontanae*	2	0	0	0	0	0	0	2
*Nasutitermesaduncus*	2	1	0	0	0	0	0	3
*Nasutitermesbrevioculatus*	0	2	0	0	1	0	0	3
*Nasutitermescolimae*	2	0	0	1	0	0	0	3
*Nasutitermescrassus*	2	1	0	0	0	0	0	3
*Nasutitermesglabritergus*	2	1	0	0	0	0	0	3
*Nasutitermesjaraguae*	2	1	0	0	0	0	0	3
*Nasutitermesmaximus*	2	1	0	0	0	0	0	3
*Nasutitermesmyersi*	2	1	0	0	0	0	0	3
*Nasutitermesnordenskioldi*	2	1	0	0	0	0	0	3
*Nasutitermesperuanus*	2	1	0	0	0	0	0	3
*Nasutitermespictus*	2	0	0	1	0	0	0	3
*Nasutitermespilosus*	2	1	0	0	0	0	0	3
*Nasutitermessanctaeanae*	2	1	0	0	0	0	0	3
*Nasutitermestipuanicus*	2	1	0	0	0	0	0	3
*Nasutitermesarenarius*	1	2	0	0	1	0	0	4
*Nasutitermesbivalens*	2	1	0	0	1	0	0	4
*Nasutitermesbolivianus*	2	2	0	0	0	0	0	4
*Nasutitermesdendrophilus*	2	2	0	0	0	0	0	4
*Nasutitermesecuadorianus*	2	2	0	0	0	0	0	4
*Nasutitermesitapocuensis*	2	2	0	0	0	0	0	4
*Nasutitermeslongiarticulatus*	2	1	1	0	0	0	0	4
*Nasutitermeslongirostratus*	2	2	0	0	0	0	0	4
*Nasutitermesmajor*	2	2	0	0	0	0	0	4
*Nasutitermesminor*	2	2	0	0	0	0	0	4
*Nasutitermesmojosensis*	2	2	0	0	0	0	0	4
*Nasutitermestredecimarticulatus*	2	2	0	0	0	0	0	4
*Nasutitermesbolivari*	2	2	1	0	0	0	0	5
*Nasutitermeschaquimayensis*	2	2	0	0	1	0	0	5
*Nasutitermescomstockae*	2	1	1	1	0	0	0	5
*Nasutitermesehrhardti*	2	2	0	0	0	1	0	5
*Nasutitermesglobiceps*	2	2	0	0	1	0	0	5
*Nasutitermeshubbardi*	2	1	0	0	1	1	0	5
*Nasutitermesminimus*	2	2	0	0	1	0	0	5
*Nasutitermespluriarticulatus*	2	2	0	0	1	0	0	5
*Nasutitermesproximus*	2	2	0	0	1	0	0	5
*Nasutitermesrotundatus*	2	1	0	0	1	1	0	5
*Nasutitermestatarendae*	2	1	0	1	1	0	0	5
*Nasutitermesaraujoi*	2	1	1	1	1	0	0	6
*Nasutitermescallimorphus*	2	1	1	1	0	0	1	6
*Nasutitermesllinquipatensis*	2	2	1	1	0	0	0	6
*Nasutitermesmacrocephalus*	0	2	1	1	1	1	0	6
*Nasutitermesmaniseri*	2	1	1	1	1	0	0	6
*Nasutitermesobscurus*	2	1	0	1	1	0	1	6
*Nasutitermesrippertii*	0	2	1	1	1	1	0	6
*Nasutitermesstricticeps*	2	1	1	1	1	0	0	6
*Nasutitermesunduliceps*	2	1	1	1	0	0	1	6
*Nasutitermesacangussu*	2	1	1	1	1	0	1	7
*Nasutitermesbanksi*	2	2	1	1	1	0	0	7
*Nasutitermesgaigei*	2	1	1	1	1	1	0	7
*Nasutitermesnigriceps*	0	2	1	1	1	1	1	7
*Nasutitermesoctopilis*	2	1	1	1	1	0	1	7
*Nasutitermeswheeleri*	2	1	1	1	1	0	1	7
*Nasutitermesacajutlae*	2	1	1	1	1	1	1	8
*Nasutitermesdasyopsis*	2	2	1	1	1	0	1	8
*Nasutitermesguayanae*	2	2	1	1	1	0	1	8
*Nasutitermeskemneri*	2	2	1	1	1	0	1	8
*Nasutitermessimilis*	2	2	1	1	1	0	1	8
*Nasutitermessurinamensis*	2	2	1	1	1	0	1	8
*Nasutitermesaquilinus*	2	2	1	1	1	2	0	9
*Nasutitermescorniger*	1	2	1	1	1	2	1	9
*Nasutitermescoxipoensis*	2	2	1	1	1	1	1	9
*Nasutitermesephratae*	2	2	1	1	1	1	1	9

- 0: Irresolvable;
- 1–4: The species identity is confusing, identifications with this name are doubtful and a revision and redescription of the type specimen(s) is necessary;
- 5–7: The identification is possible by a specialist through comparison with reference material (type specimens or specimens identified by direct comparison with types);
- 8–10: The identification is possible by a specialist with literature references.

## ﻿Results

Of the 66 species evaluated, none scored 0, 30 scored 1–4, 26 scored 5–7, 10 scored 8–10, and none scored 10 (Table 1).

### ﻿*Nasutitermesacajutlae* (Holmgren, 1910)

The identity of this species relative to *N.nigriceps* was debated for a long time. Thorne et al. (1994) summarized the taxonomic discussion and, based on a few morphological characters of the soldiers and biogeographical data (including an erroneous assumption) from the examined material, considered the two species valid. In a subsequent paper, Thorne et al. (1996) clarified the misunderstanding about the distribution of *N.acajutlae* and the resulting confusion regarding the localities of the samples examined in their previous paper. They concluded that Holmgren’s imagoes from Acajutla are probably *N.nigriceps*, closely related to *N.acajutlae*, and that at this point, the imagoes of both species are indistinguishable. They also determined that the syntype series of *N.acajutlae* includes two species. To correct the earlier mistake, they designated the soldier from Holmgren’s St. Thomas syntype series as the lectotype of *Nasutitermesacajutlae*.

Scheffrahn et al. (2005a, 2005b) and Roy et al. (2014) performed an identity assessment of the species based on DNA. Scheffrahn et al. (1994, 2003) made a good compilation of records for this species in the Antilles, but the records for the continent were not compiled. Perhaps this case deserves another deeper examination of all the material of both species *N.nigriceps* and *N.acajutlae*.

### ﻿*Nasutitermesacangussu* Bandeira & Fontes, 1979

There is only the original illustration and description (including soldiers and workers). As for other Amazonian species, the known distribution is limited. There is no map with full records from collections. Nonetheless, the species was not recorded outside the Amazon, and as it is easily identified, it is probably restricted to this biome. Cuezzo et al. (2017) provided images of the worker enteric valve. Roy et al. (2014) performed an identity assessment based on DNA.

### ﻿*Nasutitermesaduncus* Snyder, 1926

The description is poor and the only available illustration (a soldier) is a simple one. The workers are mentioned in the description, but without details.

### ﻿*Nasutitermesaquilinus* (Holmgren, 1910)

The species was redescribed by Fontes and Terra (1981) and many later papers provide information on its nest, biology, and distribution. The compilation of records in Fontes and Terra (1981) and from Torales et al. (2005) gives an overview of the species’ distribution.

### ﻿*Nasutitermesaraujoi* Roonwal & Rathore, 1976

The original description is quite complete, although the validity of the species is debatable [Constantino (2002b) points out it may be a synonym of *N.corniger*]. There are no photos of the species, although the original illustrations are detailed.

### ﻿*Nasutitermesarenarius* (Hagen, 1858)

Hagen (1858) described the imago, soldier, worker, and nymphs from Santarém, Pará State. In fact, Hagen reported Bates’ description. Holmgren (1910) copied Hagen’s description of the imago ipsis litteris, and added his own description of the soldier and worker, with a figure (Holmgren 1910: fig. 43). Holmgren (1910) reported this species from two localities: Santarém, Pará, Brazil (Bates, ex-Hagen) and Coxipó, Mato Grosso, Brazil (from Silvestri 1903). Holmgren affirmed that it is questionable whether the specimens described by Silvestri as *Eutermesarenarius* were indeed of the same species described by Bates. This challenge could only be solved after comparison of their respective material, which he did not do.

Snyder mentioned Holmgren’s (1910) work under this species in Snyder (1949) catalog, but considered only imagoes from Santarém as *N.arenarius*; Holmgren’s worker and soldier were considered *N.kemneri*, a new name applied by them.

There are no figures of the nest, but in the original description Hagen (1858), mentioned that they are formed of polycalic constructions in the soil, covering some clumps of vegetation.

### ﻿*Nasutitermesbanksi* Emerson, 1925

Constantino (1991) gave a good redescription of the species. There is no nest description, just some notes on the microhabitat where the species is found; it probably does not construct any defined nest and lives in diffuse galleries. Cuezzo et al. (2017) provided images of the worker enteric valve. Although there is no compilation of species records, several papers mention its occurrence.

### ﻿*Nasutitermesbivalens* (Holmgren, 1910)

The soldier caste dimorphism was originally the main diagnostic character used to delimit this species, but this character can easily induce an error relative to intracolonial size variation in samples, especially true for small samples. The original illustrations by Holmgren are simple and uninformative. Milano and Fontes (2002) provided photos of nests attributed to this species and included a map, but the species identification is dubious and the compilation of occurrences is not helpful.

### ﻿*Nasutitermesbolivari* (Snyder, 1959)

The soldier was illustrated by Mathews (1977), and because the species was originally described in the genus *Velocitermes*, without illustration, the original diagnosis does not include comparisons with other species of *Nasutitermes*.

### ﻿*Nasutitermesbolivianus* (Holmgren, 1910)

The original description is short and made in comparison with *N.chaquimayensis* and *N.peruanus*. Holmgren (1910) indicated that *N.bolivianus*, *N.chaquimayensis*, *N.peruanus*, *N.tambopatensis*, and *N.mojosensis*, all described by him, may be races of the same species. Accordingly, these may all be synonyms but this necessitates a revision of the species concepts and characters for each of these names.

### ﻿*Nasutitermesbrevioculatus* (Holmgren, 1910)

Krishna et al. (2013) mentioned that the syntype series of this species may be a mixture of species. The species description is generic and unhelpful. Torales et al. (2005) mapped some records for Argentina, but the identifications are dubious.

### ﻿*Nasutitermescallimorphus* Mathews, 1977

Mathews’ description and illustrations are reasonable and include data about the micro-habitats where the species is found, in an abandoned nests and another group of workers and soldiers from a covered runway in a Gallery Forest. Roy et al. (2014) performed an identity assessment based on DNA.

### ﻿*Nasutitermeschaquimayensis* (Holmgren, 1906)

Holmgren (1906) provided a discussion about the distribution of this species and its disjunction relative to other species of *Nasutitermes*, but the morphological comparison was made relative to “*Eutermesripperti*”(= *Eutermes Ripperti* f. Ehrhardti”, in Holmgren 1910: 276), a taxon whose identity is confused. Holmgren (1910) redescribed the imagoes, soldiers, and workers, with illustrations of all castes. See notes under *N.bolivianus*.

### ﻿*Nasutitermescolimae* Light, 1933

This species was described based only on imagoes (just two individuals), and the delimitation was made solely in comparison with other species described in the same work (*N.pictus*), also described only from imagoes. The species has never been recorded since the original description.

### ﻿*Nasutitermescomstockae* Emerson,1925

The original description is reasonably detailed, but the species has been mentioned only in catalogs since then. Cuezzo et al. (2017) provided images of the worker enteric valve. The soldier of this species is similar to *N.surinamensis* (EMC pers. obs.).

### ﻿*Nasutitermescorniger* (Motschulsky, 1855)

There are abundant data for this species, including DNA population studies. One of the major problems with the taxonomy of this species is that its distribution overlaps with practically all other species of the genus in the New World. Moreover, it can be misidentified with some similar species (e.g., *N.ephratae* and *N.coxipoensis*), and many other species names are probably synonyms. A reliable identification requires a sample with a good number of individuals and, preferably, data on the nest. There are some suggestions from taxonomists (personal information) that *N.globiceps*, *N.tatarendae*, and *N.araujoi* may be synonyms of *N.corniger*, but this need to be investigated. The imagoes described by Banks (1918) are not from the type series, but he made the description with Motschlsky specimens in hands.

### ﻿*Nasutitermescoxipoensis* (Holmgren, 1910)

The redescription of this species by Mathews (1977) provided more detailed information about nesting and biology. Holmgren (1910) described this species based on samples considered by Silvestri as a “Eutermesarenariussubsp.proximus var. β”, Silvestri described the queen and king, but Holmgren did not mention the imagoes in his *N.coxipoensis* description. Nonetheless, the identification of this species remains difficult, and it can easily be misidentified with other close species. For an accurate identification a good number of specimens and preferably nest data are needed. Species identity was assessed based on DNA by Roy et al. (2014). Torales et al. (2005) mapped records for Argentina, but for the remainder South America such occurrences are spread across the literature and have yet to be compiled.

### ﻿*Nasutitermescrassus* Snyder, 1926

The species has a poor description and the species has been mentioned only in catalogs since then.

### ﻿*Nasutitermesdasyopsis* Thorne, 1989

The original description (Thorne and Levings 1989) is precise and based on all castes. It is also well illustrated, with a photograph of the nest. In addition, it was mentioned in papers with molecular data, but the species has been sparsely cited in the literature. The COII sequence in GenBank DQ442177 generated by Inward et al. (2007) is 99.27% identical to *N.nigriceps*, which suggested it could be junior synonym of *N.nigriceps*. (R. Scheffrahn information), so is desirable to verify the possibility of synonymy.

### ﻿*Nasutitermesdendrophilus* (Desneux, 1906)

Desneux described the three castes without illustrations and the description is currently insufficient for proper identification. Desneux mentioned that this is a “subspecies of *T. Ripperti* Rambur”, and the imagoes were similar, while the soldiers were different. Holmgren (1910) re-described imagoes, soldiers, and workers, including some simple figures. Although both descriptions give some measurements, which can help, some of them are today uninformative. Krishna et al. (2013) cited material at the AMNH and the United States National Museum (USNM) as syntypes without any further information.

### ﻿*Nasutitermesecuadorianus* (Holmgren, 1910)

Holmgren described it originally as a “forma” (in the taxonomic sense) of *N.peruanus*. The species has been mentioned only in catalogs or species lists since the original description. Following Holmgren’s (1910) concepts mentioned in his introduction, we may consider that all those designated by him as “forma” are probably synonyms of the mentioned species (in this case, *N.peruanus*). See notes under *N.bolivianus*.

### ﻿*Nasutitermesehrhardti* (Holmgren, 1910)

The syntypes series are hosted in five different institutions, and become from distinct collection sites (Krishna et al. 2013), so it would be necessary revise these specimens. Although Holmgren’s species delimitation is not clear, it could be identified by the description of the soldier’s pilosity as it differs from other species from the same region (states of Santa Catarina and Rio Grande do Sul, Brazil), such as *N.jaraguae*. This species is mentioned in catalogs and a few surveys. Torales et al. (2005) mapped records for Argentina, but for the remainder of South America occurrences are spread across the literature.

### ﻿*Nasutitermesephratae* (Holmgren, 1910)

Krishna et al. (2013) took three pages to summarize the works that referenced this species, which reflect the large number of studies involving it using different biological approaches. Recently, a phylogeographic study of this species in the Neotropics was published (Santos et al. 2022). This species was described by Holmgren (1910) based on imagoes; the first soldier description was made by Banks (1918), but it was not based on type material. In addition, the specimens used in the work are not from the type locality and the figures are poor. Most authors follow the species delimitation made by Emerson (1925). Occurrences are listed in several works but have never been compiled.

### ﻿*Nasutitermesfeytaudi* (Holmgren, 1910)

Holmgren noted that, “it is very similar to *N.itapocuensis*, although considerably smaller, almost consonant with it” (see notes under *N.itapocuensis*). After the original description, based only on imagoes, this species was cited only in catalogs. Probably a synonym of *N.jaraguae* (EMC pers. obs.).

### ﻿*Nasutitermesgaigei* Emerson, 1925

There is some information on this species in the literature, but it has not been compiled. Based on personal observations (EMC pers. obs.), this species should be removed from Nasutitermes and placed into a new genus. However, this reclassification requires further investigation and formal study. The imago and soldiers described are from the same type series (Emerson 1925).

### ﻿*Nasutitermesglabritergus* Snyder & Emerson, 1949

The species does not have a formal description, only a mention in Snyder’s (1949) catalog of one character that made it distinct from *N.rotundatus* (absence of setae on the tergites). Nickle and Collins (1992) give a partial diagnosis for the species, although it is not clear if they consulted any type specimens.

### ﻿*Nasutitermesglobiceps* (Holmgren, 1910)

The original description is not precise and the illustrations are simple. Holmgren mentioned that the species is similar to *N.meinerti* from Venezuela, but the soldiers are larger and the nasus shorter and broader (characters that have a large intraspecific variation). Costa-Leonardo (2000) made a description of the nest and reported on the biology, but it is not clear how the species was identified. This species is possibly synonymous with *N.corniger* (see notes for *N.corniger*). Krishna et al. (2013), affirm that part of the specimens described by Silvestri (1901, 1903) as *Eutermesrippertii* are in true *N.globiceps* (Holmgren descriptions not include the imagoes, but Silvestri’s do), so we count the imago caste as described, anyway, is very desirable clarify this situation in a proper investigation.

### ﻿*Nasutitermesguayanae* (Holmgren, 1910)

Since the original description more data on the species have been collected by several authors (see Krishna et al. 2013). This includes the (indirect) characterization of the species by associated termitophiles (Emerson 1935). The imagoes are described after the original description (Emerson 1925, 1935; Adamson 1940), based on non-type specimens.

### ﻿*Nasutitermeshubbardi* Banks, 1919

The only description for this species is the original (Banks 1919). The nest was briefly mentioned (epigeal in the soil), and available figures are poorly detailed. Cuezzo et al. (2017) provided images of the worker enteric valve. The morphology of this species indicate that it is not related with the Neotropical “Nasutitermes-group” indicated by Bourguignon et al. 2017, and should be transferred to another genus, or placed in a new one (Dr. Carolina Cuezzo, IER (Conicet-UNT), Argentina, pers. comm.), a dedicated investigation is required to solve this question in the future.

### ﻿*Nasutitermesitapocuensis* (Holmgren, 1910)

Krishna et al. (2013) reported some series of syntypes whose localities are all from the State of Santa Catarina, Brazil, except for one from São Paulo State, Brazil, housed in different institutions. The species description is reasonable, although the figures are poor. E.M.C. has examined the types from the Holmgren collection at the NHRM, and it seems to be synonymous with *N.jaraguae* (the proper taxonomic and nomenclatural changes will be done elsewhere).

### ﻿*Nasutitermesjaraguae* (Holmgren, 1910)

This species was described based on soldiers and workers, and the characters are similar to those of *N.itapocuensis*, differing by soldier size. It is under study at our laboratory and molecular data suggest that this is part of a species complex (Barbosa 2018).

### ﻿*Nasutitermeskemneri* Snyder & Emerson, 1949

Snyder and Emerson (in Snyder 1949) gave this name to specimens from Coxipó, Mato Grosso, Brazil, that had been previously misidentified by Silvestri (1901) as “*Eutermesarenarius*”. However, they did not redescribe it; they only mentioned Silvestri’s text, which while validating the name left it confused. Mathews (1977) made a good redescription of this species and added some data about its biology.

### ﻿*Nasutitermeslividus* (Burmeister, 1839)

The description is based on imagoes only, the soldier caste is unknown. The types at the MCZ are pinned and are basically uninformative.

### ﻿*Nasutitermesllinquipatensis* (Holmgren, 1906)

Holmgren (1906, 1910) presented only simple illustrations; the species has been mentioned nearly only in catalogs since it was described.

### ﻿*Nasutitermeslongiarticulatus* (Holmgren, 1910)

The description of the soldier (Silvestri 1903) is brief and the only illustration of the species is a poorly detailed plate in the same work. The species has been mentioned only in catalogs since it was described.

### ﻿*Nasutitermeslongirostratus* (Holmgren, 1906)

The original description was based on the soldier and worker with simple illustrations (Holmgren 1906: figs W^1^, X^1^; 1910: 208). The imago was described by the same author, but later (Holmgren 1910). The author included it in the subgenus Subulitermes with the description and illustration of the imago and soldier (Holmgren 1910: 300, fig. 69). In fact, this species appear does not belong to *Nasutitermes*, which can be suspected even by examining the simple figures from Holmgren. A deeper investigation is necessary to affirm whether it is a species of *Subulitermes*.

### ﻿*Nasutitermesmacrocephalus* (Silvestri, 1903)

Described originally as a subspecies of *Eutermesrippertii*. The syntype series appears to be a mixture of species (Krishna et al. 2013). Most works with information about biology follow Holmgren’s (1910) species delimitation, which includes a simple figure but with distinctive characters. Cuezzo et al. (2017) provided images of the worker enteric valve. Torales et al. (2005) mapped some records for Argentina, but for other countries occurrences are scant.

### ﻿*Nasutitermesmajor* (Holmgren, 1906)

The original description and illustrations are poor, and the species has been mentioned only in catalogs since then. The imago was described by the same author, but later (Holmgren 1910).

### ﻿*Nasutitermesmaniseri* (John, 1920)

Krishna et al. (2013) mentioned a personal note of Emerson that this species is a probable synonym of *N.globiceps*. Nevertheless, the species description mentions microscopic setae on the head, absent in *N.globiceps*, which disagrees with Emerson’s observation (EMC pers. obs.). The nest was described as brown and arboreal, 3 m from the ground. The species has been mentioned only in catalogs since the original description.

### ﻿*Nasutitermesmaximus* (Holmgren, 1910)

Holmgren (1910) considered that *Eutermesmajor* (currently *N.major*) was similar to this species, but larger. The locality given by Holmgren is the same for both: Chaquimayo, Peru. The description of both species suggests they are synonymous. *Nasutitermesmaximus* has only been mentioned in catalogs since the original description.

### ﻿*Nasutitermesmeinerti* (Wasmann, 1894)

The original description was a footnote that mentions only the worker, but Holmgren (1910) included a description and illustration of the soldier based on Wasmann’s samples, although the figures and description are poor in details. The type specimens are pinned workers. See the notes for *N.globiceps*, above.

### ﻿*Nasutitermesminimus* (Holmgren, 1906)

Holmgren (1910) considered that the species as similar to *N.chaquimayensis* but even smaller, lighter, and with the same pilosity. There is a description of color, which is not useful, as it is the same for many species of *Nasutitermes*. The locality given by Holmgren is the same of *N.chaquimayensis*, Chaquimayo, Peru, besides San Firmino, Bolivia. He indicated that this species may be a, “race of *N.chaquimayo*”, although the nest is quite different. Again, it seems both may be synonymous.

### ﻿*Nasutitermesminor* (Holmgren, 1906)

Holmgren’s (1910) description is short, but it could be useful in comparison with other species as he describes the pilosity, which in this case is quite peculiar. He mentions a thin head covered by microscopic setae and a few long bristles at the base of the nasus and vertex, and he also provided an account of the shape of the head and nasus and measurements, with a simple illustration. Snyder (1926) described the imago, but the description is not that helpful.

### ﻿*Nasutitermesmojosensis* (Holmgren, 1910)

Krishna et al. (2013) mentioned a personal note of Emerson that this species is a probable synonym of *N.chaquimayensis*. Holmgren’s description is not helpful to recognize it. The species has been mentioned only in catalogs since the original description. See notes under *N.bolivianus*.

### ﻿*Nasutitermesmontanae* (Holmgren, 1910)

Described by the imago and mentioned only in catalogs since its original description. The locality is Montana, Suriname.

### ﻿*Nasutitermesmyersi* Snyder, 1933

Snyder mentioned it as, “close to *N.macrocephalus*”. The species has been mentioned only in catalogs since the original description.

### ﻿*Nasutitermesnigriceps* (Haldeman, 1854)

A large number of works referred to this species. However, the identifications follow the diagnosis of Light (1933), which included the imago description and was provided well after the original description, the types are almost certainly lost (Krishna et al. 2013). Like *N.corniger*, it is a widespread species (Dr. R. Rudolf H. Scheffrahn, University of Florida, U.S., pers. comm.). Distribution records are scattered through the literature.

### ﻿*Nasutitermesnordenskioldi* (Holmgren, 1910)

Holmgren’s description does not mention clear distinctive characters for this species. Nevertheless, the shape, size, and peculiar pilosity, allied to localities mentioned by Holmgren, may help an expert to solve the species’ identity. The species has been mentioned only in catalogs since the original description.

### ﻿*Nasutitermesobscurus* (Holmgren, 1906)

This species was renamed by Snyder and Emerson (in Snyder 1949) as *Nasutitermeslighti*, an action that was dropped by Krishna et al. (2013), who reverted it to the previous name. Krishna et al. (2013) mentioned that the syntypes at the AMNH agree well with the description in Holmgren (1910). Nevertheless, they also mention a note by Emerson considering that the original description (Holmgren 1906) may be based on two different species. Regardless, the existing species description is too generic. The nest is described as an arboreal carton. Roy et al. (2014) characterized the species by DNA.

### ﻿*Nasutitermesoctopilis* Banks, 1918

The species is well characterized, it can easily be identified by Emerson’s (1925) description (that included the imago), and it has been characterized by DNA data (Roy et al. 2014). Cuezzo et al. (2017) provided images of the worker enteric valve. There is no description of the nest and field notes suggest that this species lives in diffuse galleries in the soil and dead wood (Constantino 1991).

### ﻿*Nasutitermesperuanus* (Holmgren, 1910)

Holmgren described the soldier of this species as, “very similar to *N.chaquimayensis*, but larger”, which makes this diagnosis even more problematic since the identity of *N.chaquimayensis* is unclear. The soldier illustration is simple and the species has been mentioned only in catalogs since the original description. See notes under *N.bolivianus*.

### ﻿*Nasutitermespictus* Light, 1933

Nickle and Collins (1990) provided figures of this species but the species was described based only on imagoes (see notes under *N.colimae*). It has never again been recorded since the original description.

### ﻿*Nasutitermespilosus* Snyder, 1926

The original description characterized this species relative to *N.cayennae* (considered a junior synonym of *N.corniger*), with few characters and without illustrations. The species has been mentioned only in catalogs since the original description.

### ﻿*Nasutitermespluriarticulatus* (Silvestri, 1901)

Holmgren’s (1910) illustrations are simple but they clearly differ from Silvestri’s (1903) plates. Silvestri (1903) mentions the nesting and feeding habits of this species as the, “same as *N.proximus*”, another taxon of dubious identity.

### ﻿*Nasutitermesproximus* (Silvestri, 1901)

Holmgren (1910) provided a simple illustration of the soldier. Silvestri (1903) gave a poor illustration of the imago and mentioned the nest and feeding habits of this species as the “same as of *Eutermesarenarius*” (samples that which were later renamed as *N.kemneri*).

### ﻿*Nasutitermesrippertii* (Rambur, 1842)

The species was originally described based on the imago. Later, Banks (1919) synonymized *Eutermescubanus* and *Eutermesbahamensis* [both described by Holmgren (1910)] with *N.rippertii*, and the species diagnosis (including the soldier description) came from this material determined by Banks. Since then, some works have increased the available data for the species. Scheffrahn et al. (2006) made a compilation of records in the Antilles. The holotype is probably lost. The Institut Royal des Sciences Naturelles de Belgique (RIB) online catalog (RBINS Virtual Collections 2023) does not list this specimen in their collection. If the type exists, it is certainly pinned, as is the case for other material from Rambur.

### ﻿*Nasutitermesrotundatus* (Holmgren, 1906)

The species illustration is simple (Holmgren 1910) and its delimitation was made in comparison with *Eutermesrobustus* (currently *Sandsitermesrobustus*). Bandeira and Vasconcellos (2002) related that the species lives and feeds in wood, and the MZUSP field notes reinforce that this species does not build a conspicuous nest. Moreira et al. (2008) provided a detailed study of the worker gut. Torales et al. (2005) mapped records in Argentina but occurrences for other countries remain fragmented.

### ﻿*Nasutitermessanctaeanae* (Holmgren, 1910)

Holmgren described this species based on Silvestri’s (1903) specimens determined as a variety “alpha” of *Eutermesarenariusproximus*. There are no illustrations and the species has been mentioned only in catalogs since the original description. Torales et al. (2005) listed this species for Argentina, but the record refers only to Silvestri’s specimens.

### ﻿*Nasutitermessimilis* Emerson, 1935

Emerson’s characterization of this species included the associated termitophiles, which were distinct from those of *N.guayanae* (a morphologically close species). Cuezzo et al. (2017) provided images of the worker enteric valve. Roy et al. (2014) characterized the species by DNA.

### ﻿*Nasutitermesstricticeps*Mathews (1977)

Mathews made a detailed description of this species but it hasn’t been registered again since the original description. Cuezzo et al. (2017) provided images of the worker enteric valve.

### ﻿*Nasutitermessurinamensis* (Holmgren, 1910)

The imago was described latter, by Emerson (1925), based on non-type specimens. Mathews (1977) published photographs and described the species’ nest. Roy et al. (2014) characterized the species by DNA. Cuezzo et al. (2017) provided images of the worker enteric valve.

### ﻿*Nasutitermestatarendae* (Holmgren, 1910)

Holmgren (1910) mentioned the soldier as, “being very similar to *N.major*, but darker”. Mathews (1977) redescribed this species and some additional biological data were subsequently published. However, several authors indicate that this species is a probable synonym of *N.corniger*. See notes under *N.bolivianus*.

### ﻿*Nasutitermestipuanicus* (Holmgren, 1910)

The original description characterizes this species as similar to *N.pluriarticulatus*, a species of dubious identity (see above). The curator of the ZMH confirmed that the types are preserved in alcohol. There are no illustrations of the species and it has only been mentioned in catalogs since the original description.

### ﻿*Nasutitermestredecimarticulatus* (Holmgren, 1910)

Holmgren described the soldier as, “nearly identical to *Eutermesmajor*” (currently *N.major*; see the comments about this species above), without more detailed information. There is only an illustration of the imago and the sole record of the species apart from the original description was made by Bandeira and Macambira (1988), with notes on its feeding habits and occurrence in different vegetative habitats.

### ﻿*Nasutitermesunduliceps* Mathews, 1977

Mathews made a complete description of the soldier, with a simple illustration of its head. Roy et al. (2014) characterized the species by DNA.

### ﻿*Nasutitermeswheeleri* Emerson, 1925

Emerson’s illustrations show some distinctive characters of this species. Mathews (1977) published photographs and described the nest. Cuezzo et al. (2017) provided images of the worker enteric valve. Roy et al. (2014) characterized the species by DNA.

## ﻿Discussion

### ﻿About the criteria and their scores

We intentionally tried to keep the criteria evaluations as restrictive as possible. It is clear that some criteria are more easily recognized as discrete, while others are more continuous. Type specimens and the castes used for description can be comfortably accommodated within the listed options, but evaluating species descriptions and illustrations is challenging due to potential subjectivity biases.

Opting for fewer categories was a tentative approach to maintain clarity. We recognize that intermediate situations exist for descriptions and illustrations. In practice, they follow a progressive scale of “obsolescence”, with older descriptions failing to consider characters discussed in more recent descriptions and thus becoming progressively outdated. Our practical solution was to maintain a few states for these cases.

The same applies to information about biology. The quality and type of information about each species are certainly very heterogeneous. However, trying to qualify all types of information would significantly increase the workload for assessments with little return. This is why we have included a list of notes to complement the information.

The ranks are intended to assess the viability of species identity for taxonomic studies and establish priorities for resolution, rather than to evaluate the robustness of species identifications. We aim for an operational response by describing what we consider informative. In this way, if the criteria contribute to the robustness of the species hypothesis, the score is achieved.

### ﻿*Nasutitermes* results for species

It is necessary to reiterate that species of *Nasutitermes* are challenging to identify, even when good descriptions do exist. In several cases a reliable identification is impossible without direct comparison with type specimens or material previously compared against types. Ultimately, this may be impossible for many species under currently available data.

Comparison with determined material in any collection should be undertaken with caution, because not all samples in a collection, even a well-curated one, are correctly identified. It is necessary to know who has determined them and at what time (as species concepts can change over time). Any examiner should be critical as to when the material was determined and if more data for the species has since been accumulated. The best is to compare with types, or if lacking, to search for material compared with types by an expert researcher (typically referred to as a “metatype” by Emerson, although it should be noted that such specimens hold no nomenclatural standing under the ICZN). Sometimes a curator may identify specimens even tentatively, considering that it is better than to leave the sample in a mass of “unidentified material”. It is a common practice and a non-specialist should be cautious doing this.

Nearly half of the species evaluated scored between 1 and 4 (i.e., 30 species were considered “confusing”), and the majority of these cases (24) consisted of species that scored only in the “Type specimens” and/or “Castes used for description” criteria; none of the species were considered irresolvable.

Low-ranked species have been recurrently cited only in catalogs after the original description, which suggests that some of these names are synonyms, but this cannot be used as a rule. It became evident that species checklists based on literature records extend the longevity of these “taxonomically confusing species’’ in databases, which implies some noise for biodiversity evaluations and certainly is problematic for researchers who only mine data rather than verify it from the original sources.

The majority of these low-ranked species have type specimens and it is therefore imperative to clarify their identity as this will allow for advances in understanding these species.

Among the species with a total score comprised between 5 and 9, the most relevant criteria, after the “Type specimens” and “Castes used for description”, are the accuracy of descriptions and illustrations, and the existence of information about the species’ biology. This is expected since the compilation of records is possible only after the accumulation of good numbers of correctly determined samples in collections, and the identification is dependent on well characterized species. This can be observed more clearly in the species with a total score comprised between 5 and 7, the presence of Information about biology is the more frequent criteria that pulls up the scores.

No species scored 10. Among the highest scores (8 and 9), the absence of information on the distribution of the species is the most frequent criterion that reduces the scores. This makes sense, as most of the *Nasutitermes* species in the collections have not been compiled and plotted on a map. Perhaps in the near future this gap will be reduced, as many databases are currently in progress.

Some peculiar cases are interesting to mention, specifically *N.corniger*, *N.macrocephalus*, *N.nigriceps*, and *N.rippertii.* These species scored low in the type criterion (types lost or badly conserved) and high in the remaining criteria. In these cases, the species identity is anchored only on redescriptions published long after the original description.

DNA studies are prevalent in species of higher taxonomic marks, as expected. As DNA sequencing becomes more accessible and cost-effective, there has been a widespread misconception regarding the utility of these data for taxonomy, particularly the notion that DNA can facilitate “rapid taxonomy”. Some of this misconception is based on the widespread perception that taxonomy was not “integrative” before DNA data become available. However, even since Darwin’s time when he demonstrated strong relationships between distribution, life habits, and morphology in species of the Galapagos Islands, suggesting distinct evolutionary lineages, taxonomy has been integrative. Nevertheless, it took considerable time for evolutionary concepts to be widely incorporated into taxonomic practices. Molecular data is the latest tool to become available for investigation and has been extensively utilized.

The primary significance of DNA data in taxonomy lies in providing a new set of characters to test species hypotheses and more inclusive groupings, specifically to test the monophyly of genera and higher taxonomic ranks. It is expected that prior to the use of DNA data for species hypothesis testing, all preceding criteria have been investigated and evolutionary lineages have been proposed. Wheeler (2004) extensively discusses these concepts, while Engel (2022) provides a concise and clear summary of these discussions.

The sequential chain of taxonomic steps, which enhances the robustness of a species hypothesis (including nomenclatural stability, species characterization, species data, and inherited characters evidencing natural lineages), becomes routine for experienced taxonomists. Gonzalez et al. (2013) elaborate on all these aspects related to the quality of taxonomic research and propose a similar cumulative sequence of operations to evaluate the status of taxonomic literature, specifically focusing on bumblebees as mentioned in the introduction.

Unfortunately, today there is an effort to reverse the taxonomic process, largely fueled by a false controversy of “morphology vs DNA”. Again, as Engel (2022) states: “A consensus barcode for any given cluster is assumed to “diagnose” the given species. In this way, hundreds of species could be wholesale sequenced for COI, clustered by BOLD, and then simply summarized by a consensus barcode sequence and given a name. Quick and easy. And yet, is it meaningful? One of the fundamental and critical roles of a taxonomist is to test species hypotheses. These rely on data, and ideally as much data as can be called upon to formulate a concept for any given species and what features, be they anatomical, behavioral, chemical, molecular, etc., serve to circumscribe that biological unit from others in nature.”

The way the criteria scores were presented (Table 1) supports a virtuous cycle of taxonomic practices. The criteria are not independent; they tend to form a ‘ladder’ in the matrix when ordered by ascending score (see in the Suppl. material 1, the numerical values of each criterion colored according to their rank).

Mathematically, various scoring combinations would be possible, but the scores, in general, do not behave independently. For example, there is no plausible scenario where two species score a total of “2”, with one species meeting only the criterion “Type specimens well-preserved (2)” and another “Data about species distribution, the species registries are compiled and mapped (2)”. The same applies to higher sums.

### ﻿Use of this protocol for other taxa

In this work, we used the Neotropical species of *Nasutitermes* as a test case. But it can be adapted to nearly any other taxon, with simple alterations to the evaluation criteria. It would be important and informative to see the results with other taxa to evaluate its broader practicality and effectiveness.

The criterion “castes used for description” makes sense primarily for social insects. However, for insects with ametabolous and hemimetabolous development, species are sometimes described based only on immature stages. It is reasonably clear that taxa with incomplete knowledge of developmental stages would have lower taxonomic health than those with more comprehensive information across various instars. The relevance of this criterion varies depending on the taxonomic practices of the group being studied.

The same applies to species described based on only one sex or form. Polymorphism within populations, such as sexual dimorphism or melanistic forms, has been a source of taxonomic confusion, leading to cases where all the species of one genus were synonymized once these gaps were understood.

In some cases, knowing the specific environment is more relevant than having highly precise geographical coordinates. For certain groups, the soil depth or the arboreal stratum in which the specimen was collected is crucial information that can help differentiate species with sympatric distributions.

The existence of voucher specimens of hosts is particularly relevant for inquiline/parasitic organisms. It is very common for the host organism to be mentioned only in the description of their inquilines/parasites. When the identity of hosts needs to be checked (e.g., when it is discovered that what was thought to be a single species of a parasite is actually two species), the information about host specimens becomes inaccessible.

Another criterion to consider is whether an evolutionary species concept was employed or if the only description available was made before the evolutionary theory consistently be accepted. This is not the case for the *Nasutitermes* species we have been working on. The first comprehensive review of Neotropical *Nasutitermes* was conducted by Holmgren in 1910. Despite the limited tools available at the time, Holmgren worked from an evolutionary perspective. Although he tended to be a “splitter” in his work and was occasionally contradictory in his critiques of Silvestri’s concepts, Holmgren reviewed almost all the material in collections up until 1900. Hagen published his treatise in 1858, one year before “On the Origin of Species”, so evolutionary concepts were not mentioned. Nevertheless, Holmgren later revised the same taxa that Hagen had worked on. Subsequent authors, most notably Emerson, Snyder, and Light, clearly worked under an evolutionary scenario.

We believe that it would be interesting to evaluate older species descriptions, particularly those that remain outdated, to see how many species concepts have been updated from a Linnean/Aristotelian framework to an evolutionary perspective.

## ﻿Additional information

### Author ORCIDs

Mauricio M. Rocha https://orcid.org/0000-0002-6568-068X

Karina S. S. Lima https://orcid.org/0000-0003-3063-1615

Eliana M. Cancello https://orcid.org/0000-0003-3125-6335

### Data availability

All of the data that support the findings of this study are available in the main text or Supplementary Information.

## Supplementary materials

Supplementary material 1 Supplementary information Data type xls https://binary.pensoft.net/file/1119124This dataset is made available under the Open Database License (http://opendatacommons.org/licenses/odbl/1.0/). The Open Database License (ODbL) is a license agreement intended to allow users to freely share, modify, and use this Dataset while maintaining this same freedom for others, provided that the original source and author(s) are credited.Mauricio M. Rocha, Karina S. S. Lima, Eliana M. Cancello

## References

[B1] Adamson AM (1940) New termite intercastes. Proceedings of the Royal Society of London. Series B, Biological Sciences 129(854): 35–53. 10.1098/rspb.1940.0028

[B2] Bandeira AG Fontes LR (1979) *Nasutitermesacangussu*, a new species of termite from Brazil (Isoptera, Termitidae, Nasutitermitinae). Revista Brasileira de Entomologia 23(3): 119–122.

[B3] Bandeira AG Macambira MLJ (1988) Térmitas de Carajás, Estado do Pará, Brasil: composição faunística, distribuição e hábito alimentar. Boletim do Museu Paraense Emílio Goeldi, Série Zoologia 4(2): 175–190. https://repositorio.museu-goeldi.br/handle/mgoeldi/404

[B4] Bandeira AG Vasconcellos A (2002) A quantitative survey of termites in a gradient of disturbed highland forest in northeastern Brazil (Isoptera). Sociobiology 39(3): 429–439.

[B5] Banks N (1918) The termites of Panama and British Guiana. Bulletin of the American Museum of Natural History 38(17): 659–667 [+ 1 pl.]. http://hdl.handle.net/2246/1818

[B6] Banks N (1919) Antillean Isoptera. Bulletin of the Museum of Comparative Zoology 26: 475–489. https://www.biodiversitylibrary.org/page/2802128#page/615/mode/1up

[B7] Barbosa NCCP (2018) Integrando diferentes abordagens genéticas para a compreensão dos cupins: delimitação de espécies do Grupo *Nasutitermesjaraguae* (Holmgren, 1910) (Isoptera: Termitidae: Nasutitermitinae), filogeografia da espécie *N.jaraguae* no bioma Mata Atlântica e filogenia das espécies do gênero *Nasutitermes* Dudley, 1890. PhD thesis, Universidade Estadual Paulista, São José do Rio Preto, Brazil. http://hdl.handle.net/11449/154344

[B8] Barnosky AD Matzke N Tomiya S Wogan GOU Swartz B Quental TB Marshall C McGuire JL Lindsey EL Maguire KC Mersey B Ferrer EA (2011) Has the Earth’s sixth mass extinction already arrived? Nature 471(7336): 51–57. 10.1038/nature09678 21368823

[B9] Bini LM Diniz-Filho JAF Rangel TFLVB Bastos RP Pinto MP (2006) Challenging Wallacean and Linnean shortfalls: Knowledge gradients and conservation planning in a biodiversity hotspot. Diversity & Distributions 12(5): 475–482. 10.1111/j.1366-9516.2006.00286.x

[B10] Bourguignon T Lo N Šobotník J Ho SY Iqbal N Coissac E Lee M Jendryka M Sillam-Dussès D Křížková B Roisin Y Evans TA (2017) Mitochondrial phylogenomics resolves the global spread of higher termites, ecosystem engineers of the tropics. Molecular Biology and Evolution 34: 589–597. 10.1093/molbev/msw253 28025274

[B11] Britz R Hundsdörfer A Fritz U (2020) Funding, training, permits – the three big challenges of taxonomy. Megataxa 1(1): 49–52. 10.11646/megataxa.1.1.10

[B12] Burmeister H (1839) Termiten, weisse Ameisen. Termitina. In: Burmeister H Handbuch der Entomologie. Vol. 2, Theod. Chr. Friedr. Enslin, Berlin, 758–768.

[B13] Constantino R (1991) Termites (Isoptera) from the lower Japurá River, Amazonas State, Brazil. Boletim do Museu Paraense Emílio Goeldi, Série Zoologia 7(2): 189–224. https://repositorio.museu-goeldi.br/handle/mgoeldi/744

[B14] Constantino R (2002a) Notes on the type-species and synonymy of the genus *Nasutitermes* (Isoptera: Termitidae: Nasutitermitinae). Sociobiology 40(3): 533–537.

[B15] Constantino R (2002b) The pest termites of South America: Taxonomy, distribution and status. Journal of Applied Entomology 126(7–8): 355–365. 10.1046/j.1439-0418.2002.00670.x

[B16] Constantino R (2018) Estimating Global Termite Species Richness Using Extrapolation. Sociobiology 65(1): 10–14. 10.13102/sociobiology.v65i1.1845

[B17] Constantino R (2023) Online termite database. http://164.41.140.9/catal/ [Accessed on 30 Oct 2023]

[B18] Costa-Leonardo AM (2000) Biological data for the Neotropical termite *Nasutitermesglobiceps* (Isoptera: Termitidae: Nasutitermitinae). Sociobiology 36(3): 63–71.

[B19] Cuezzo C Cancello EM Carrijo TF (2017) *Sandsitermes* gen. nov., a new nasute termite genus from South America (Isoptera, Termitidae, Nasutitermitinae). Zootaxa 4221(5): 562–574. 10.11646/zootaxa.4221.5.5 28187645

[B20] de Carvalho MR Bockmann FA Amorim DS Brandão CRF de Vivo M de Figueiredo JL Britski HA de Pinna MCC Menezes NA Marques FPL Papavero N Cancello EM Crisci JV McEachran JD Schelly RC Lundberg JG Gill AC Britz R Wheeler QD Stiassny MLJ Parenti LR Page LM Wheeler WC Faivovich J Vari RP Grande L Humphries CJ DeSalle R Ebach MC Nelson GJ (2007) Taxonomic impediment or impediment to taxonomy? A commentary on systematics and the cybertaxonomic-automation paradigm. Evolutionary Biology 34(3–4): 140–143. 10.1007/s11692-007-9011-6

[B21] Desneux J (1906) Variétés termitologiques. Annales de la Société Entomologique de Belgique 49(12): 336–360. https://www.biodiversitylibrary.org/page/12283977

[B22] Dirzo R Raven PH (2003) Global State of Biodiversity and Loss. Annual Review of Environment and Resources 28(1): 137–167. 10.1146/annurev.energy.28.050302.105532

[B23] Dubois A (2010) Zoological nomenclature in the century of extinctions: Priority vs. “usage”. Organisms, Diversity & Evolution 10(3): 259–274. 10.1007/s13127-010-0021-3

[B24] Eggleton P (1999) Termite species description rates and the state of termite taxonomy. Insectes Sociaux 46(1): 1–5. 10.1007/s000400050105

[B25] Emerson AE (1925) The termites of Kartabo, Bartica District, British Guiana. Zoologica (New York) 6(4): 291–459. 10.5962/p.190324

[B26] Emerson AE (1935) Termitophile distribution and quantitative characters as indicators of physiological speciation in British Guiana termites (Isoptera). Annals of the Entomological Society of America 28(3): 369–395. 10.1093/aesa/28.3.369

[B27] Engel MS (2022) The how and why of scientific naming: What’s in a name? Taprobanica 11(2): 47–53. 10.47605/tapro.v11i2.282

[B28] Engel MS Ceríaco LMP Daniel GM Dellapé PM Löbl I Marinov M Reis RE Young MT Dubois A Agarwal I Lehmann AP Alvarado M Alvarez N Andreone F Araujo-Vieira K Ascher JS Baêta D Baldo D Bandeira SA Barden P Barrasso DA Bendifallah L Bockmann FA Böhme W Borkent A Brandão CRF Busack SD Bybee SM Channing A Chatzimanolis S Christenhusz MJM Crisci JV D’elía G Da Costa LM Davis SR De Lucena CAS Deuve T Fernandes Elizalde S Faivovich J Farooq H Ferguson AW Gippoliti S Gonçalves FMP Gonzalez VH Greenbaum E Hinojosa-Díaz IA Ineich I Jiang J Kahono S Kury AB Lucinda PHF Lynch JD Malécot V Marques MP Marris JWM Mckellar RC Mendes LF Nihei SS Nishikawa K Ohler A Orrico VGD Ota H Paiva J Parrinha D Pauwels OSG Pereyra MO Pestana LB Pinheiro PDP Prendini L Prokop J Rasmussen C Rödel MO Rodrigues MT Rodríguez SM Salatnaya H Sampaio Í Sánchez-García A Shebl MA Santos BS Solórzano-Kraemer MM Sousa ACA Stoev P Teta P Trape J-F Dos Santos CV-D Vasudevan K Vink CJ Vogel G Wagner P Wappler T Ware JL Wedmann S Zacharie CK (2021) The taxonomic impediment: A shortage of taxonomists, not the lack of technical approaches. Zoological Journal of the Linnean Society 193(2): 381–387. 10.1093/zoolinnean/zlab072

[B29] Fontes LR Terra PS (1981) A study on the taxonomy and biology of the Neotropical termite *Nasutitermesaquilinus* (Isoptera, Termitidae, Nasutitermitinae). Revista Brasileira de Entomologia 25(3): 171–183.

[B30] Gonzalez VH Griswold T Engel MS (2013) Obtaining a better taxonomic understanding of native bees: Where do we start? Systematic Entomology 38(4): 645–653. 10.1111/syen.12029

[B31] Grimaldi DA Engel MS (2007) Why descriptive science still matters. Bioscience 57(8): 646–647. 10.1641/B570802

[B32] Hagen HA (1858) Monographie der Termiten. Linnaea Entomologica 12: i–iii + 4–342 + 459 + [3 pls]. https://www.biodiversitylibrary.org/page/46573554

[B33] Haldeman SS (1854) Descriptions of some new species of insects, with observations on described species. Proceedings. Academy of Natural Sciences of Philadelphia 6: 361–365. https://www.biodiversitylibrary.org/page/1779897

[B34] Holmgren N (1906) Studien über südamerikanische Termiten. Zoologische Jahrbucher. Abteilung fur Systematik, Ökologie und Geographie der Tiere 23(5): 521–676. https://biostor.org/reference/232733

[B35] Holmgren N (1910) Versuch einer Monographie der amerikanischen Eutermes-Arten. Mitteilungen aus dem Naturhistorischen Museum (Hamburg) 27: 171–325. https://biostor.org/reference/184508

[B36] Howard RW Thorne BL Levings SC McDaniel CA (1988) Cuticular hydrocarbons as chemotaxonomic characters for *Nasutitermescorniger* (Motschulsky) and *N.ephratae* (Holmgren) (Isoptera: Termitidae). Annals of the Entomological Society of America 81(3): 395–399. 10.1093/aesa/81.3.395

[B37] Inward DJ Vogler AP Eggleton P (2007) A comprehensive phylogenetic analysis of termites (Isoptera) illuminates key aspects of their evolutionary biology. Molecular Phylogenetics and Evolution 44(3): 953–967. 10.1016/j.ympev.2007.05.014 17625919

[B38] John O (1920) South American termites. Additional note to Mr. I. Strelnikov’s article on. Bulletin de L’Institut Lesshaft 1: 227–234.

[B39] Kitchener AC Hoffmann M Yamaguchi N Breitenmoser-Würsten C Wilting A (2022) A system for designating taxonomic certainty in mammals and other taxa. Mammalian Biology 102(1): 251–261. 10.1007/s42991-021-00205-3

[B40] Krishna K Grimaldi DA Krishna V Engel MS (2013) Treatise on the Isoptera of the world. Bulletin of the American Museum of Natural History 377(7): 1–2704. 10.1206/377.1

[B41] Light SF (1933) Termites of western Mexico. University of California publications in Entomology 6(5): 79–164.

[B42] Mathews AGA (1977) Studies on Termites from the Mato Grosso State, Brazil. Academia Brasileira de Ciências, Rio de Janeiro, 267 pp.

[B43] Milano S Fontes LR (2002) Cupim e cidade: implicações ecológicas e controle. Conquista Artes Gráficas, São Paulo, 141 pp.

[B44] Mora C Tittensor DP Adl S Simpson AG Worm B (2011) How many species are there on Earth and in the ocean? PLoS Biology 9(8): e1001127. 10.1371/journal.pbio.1001127 PMC3160336 21886479

[B45] Moreira J Neves CA Araújo VA Araújo APA Cruz LC Moreira PA Rocha SL (2008) Digestive system morphology of *Nasutitermesrotundatus* (Isoptera: Termitidae, Nasutitermitinae). Sociobiology 51(3): 563–578.

[B46] Motschulsky V (1855) Voyages: Lettre de M. de Motschulsky à M. Ménétriés. Etudes Entomologiques 4: 8–25.

[B47] Nickle DA Collins MS (1990) The termite fauna (Isoptera) in the vicinity of Chamela, State of Jalisco, Mexico. Folia Entomológica Mexicana 77(1988): 85–122.

[B48] Nickle DA Collins MS (1992) The termites of Panama (Isoptera). In: Quintero D Aiello A (Eds) Insects of Panama and Mesoamerica: selected studies.Oxford University Press, Oxford, US, 208–241. 10.1093/oso/9780198540182.003.0013

[B49] Pereira HM Leadley PW Proença V Alkemade R Scharlemann JPW Fernandez-Manjarrés JF Araújo MB Balvanera P Biggs R Cheung WWL Chini L Cooper HD Gilman EL Guénette S Hurtt GC Huntington HP Mace GM Oberdorff T Revenga C Rodrigues P Scholes RJ Sumaila UR Walpole M (2010) Scenarios for Global Biodiversity in the 21^st^ Century. Science 330(6010): 1496–1501. 10.1126/science.1196624 20978282

[B50] Rambur JP (1842) Histoire naturelle des insectes. Néuroptères. Librairie Encyclopédique de Roret, Paris, 534 pp.

[B51] Roonwal ML Rathore NS (1976) Termites from the Amazon Basin, Brazil, with new records and two new Nasutitermes (Insecta: Isoptera). Records of the Zoological Survey of India 69(1–4): 161–186. 10.26515/rzsi/v69/i1-4/1971/161407

[B52] Roy V Constantino R Chassany V Giusti‐Miller S Diouf M Mora P Harry M (2014) Species delimitation and phylogeny in the genus *Nasutitermes* (Termitidae: Nasutitermitinae) in French Guiana. Molecular Ecology 23(4): 902–920. 10.1111/mec.12641 24372711

[B53] Sands WA (1965) A revision of the termite subfamily Nasutitermitinae (Isoptera, Termitidae) from the Ethiopian region. Bulletin of the British Museum (Natural History). Entomology 4(Supplement. 4): 1–172. 10.5962/p.193228

[B54] Santos A Carrijo TF Cancello EM Morales-Corrêa e Castro AC (2017) Phylogeny of *Nasutitermescorniger* (Isoptera: Termitidae) in the Neotropical Region. BMC Evolutionary Biology 17(1): 230. 10.1186/s12862-017-1079-8 29169320 PMC5701342

[B55] Santos A Cancello EM Morales-Corrêa e Castro AC (2022) Phylogeography of *Nasutitermesephratae* (Termitidae: Nasutitermitinae) in Neotropical region. Scientific Reports 12(1): 11656. 10.1038/s41598-022-15407-z 35804053 PMC9270401

[B56] Scheffrahn RH Darlington JPEC Collins MS Krecek J Su NY (1994) Termites (Isoptera: Kalotermitidae, Rhinotermitidae, Termitidae) of the West Indies. Sociobiology 24(2): 213–238.

[B57] Scheffrahn RH Jones SC Křeček J Chase JA Mangold JR Su NY (2003) Taxonomy, distribution, and notes on the termites (Isoptera: Kalotermitidae, Rhinotermitidae, Termitidae) of Puerto Rico and the U.S. Virgin Islands. Annals of the Entomological Society of America 96(3): 181–201. 10.1603/0013-8746(2003)096[0181:TDANOT]2.0.CO;2

[B58] Scheffrahn RH Křeček J Szalanski AL Austin JW (2005a) Synonymy of Neotropical arboreal termites *Nasutitermescorniger* and *N.costalis* (Isoptera: Termitidae: Nasutitermitinae), with evidence from morphology, genetics, and biogeography. Annals of the Entomological Society of America 98(3): 273–281. 10.1603/0013-8746(2005)098[0273:SONATN]2.0.CO;2

[B59] Scheffrahn RH Křeček J Szalanski AL Austin JW Roisin Y (2005b) Synonymy of two arboreal termites (Isoptera: Termitidae: Nasutitermitinae): *Nasutitermescorniger* from the neotropics and *N.polygynus* from New Guinea. The Florida Entomologist 88(1): 28–33. 10.1653/0015-4040(2005)088[0028:SOTATI]2.0.CO;2

[B60] Scheffrahn RH Křeček J Chase JA Maharajh B Mangold JR (2006) Taxonomy, biogeography, and notes on termites (Isoptera: Kalotermitidae, Rhinotermitidae, Termitidae) of the Bahamas and Turks and Caicos Islands. Annals of the Entomological Society of America 99(3): 463–486. 10.1603/0013-8746(2006)99[463:TBANOT]2.0.CO;2

[B61] Silvestri F (1901) Nota preliminare sui termitidi sud-americani. Bollettino dei Musei di Zoologia e Anatomia Comparata della R. Università di Torino XVI 16(389): 1–8. 10.5962/bhl.part.26628

[B62] Silvestri F (1903) Contribuzione alla conoscenza dei Termiti e Termitofili dell’America Meridionale. Redia 1: 1–234. 10.5962/bhl.title.137413

[B63] Snyder TE (1926) Termites collected on the Mulford Biological Exploration to the Amazon Basin, 1921–1922. Proceedings of the United States National Museum 68(14): 1–76. 10.5479/si.00963801.68-2615.1

[B64] Snyder TE (1933) A new species of Brazilian termite, featuring an intermediate soldier-worker individual. Proceedings of the Biological Society of Washington 46: 161–166. https://www.biodiversitylibrary.org/page/34560895

[B65] Snyder TE (1949) Catalog of termites (Isoptera) of the world. Smithsonian Miscellaneous Collections 112(3953): 1–490.

[B66] Snyder TE (1959) New termites from Venezuela, with keys and a list of the described Venezuelan species. American Midland Naturalist 61(2): 313–321. 10.2307/2422503

[B67] Thorne BL (1980) Differences in nest architecture between the Neotropical arboreal termites *Nasutitermescorniger* and *Nasutitermesephratae* (Isoptera: Termitidae). Psyche (Cambridge, Massachusetts) 87(3–4): 235–243. 10.1155/1980/12305

[B68] Thorne BL Levings SC (1989) A new species of *Nasutitermes* (Isoptera: Termitidae) from Panama. Journal of the Kansas Entomological Society 62(3): 342–347. https://www.jstor.org/stable/25085100

[B69] Thorne BL Haverty MI Collins MS (1994) Taxonomy and biogeography of *Nasutitermesacajutlae* and *N.nigriceps* (Isoptera: Termitidae) in the Caribbean and Central America. Annals of the Entomological Society of America 87(6): 762–770. 10.1093/aesa/87.6.762

[B70] Thorne BL Haverty MI Collins MS (1996) Antillean termite named for a locality in Central America: Taxonomic memorial to a perpetuated error. Annals of the Entomological Society of America 89(3): 346–347. 10.1093/aesa/89.3.346

[B71] Torales GJ Laffont ER Godoy MC Coronel JM Arbino MO (2005) Update on taxonomy and distribution of Isoptera from Argentina. Sociobiology 45(3): 853–886.

[B72] Virtual Collections RBINS (2023) RBINS Virtual Collections. http://virtualcollections.naturalsciences.be/ [Accessed on 30 Oct 2023]

[B73] Wasmann E (1894) Kritisches Verzeichniss der myrmekophilen und termitophilen Arthropoden: Mit Angabe der Lebensweise und mit Beschreibung neuer Arten. Felix L. Dames, Berlin, Germany, 231 pp. 10.5962/bhl.title.122977

[B74] Wheeler QD (2004) Taxonomic triage and the poverty of phylogeny. Philosophical Transactions of the Royal Society of London. Series B, Biological Sciences 359(1444): 571–583. 10.1098/rstb.2003.1452 PMC1693342 15253345

[B75] Wheeler Q (2023) Species, Science and Society: The Role of Systematic Biology. 1^st^ edn. Routledge, London, 266 pp. 10.4324/9781003389071

[B76] Winston JE (1999) Describing species: Practical Taxonomic Procedure for Biologists. Columbia University Press, New York, 518 pp. https://www.jstor.org/stable/10.7312/wins06824

